# The peculiarities of food allergies in accordance with the level of injury of respiratory tract in children of Eastern Siberia

**DOI:** 10.3402/ijch.v72i0.21202

**Published:** 2013-08-05

**Authors:** Irina V. Borisova, Svetlana V. Smirnova

**Affiliations:** Federal State Budget Institution, Scientific Research Institute for Medical Problems of the North, Siberian Division of Russian Academy of Medical Sciences, Krasnoyarsk, Russia

**Keywords:** food allergy, respiratory tract, elimination diet

## Abstract

**Aim:**

To determine the course of food allergy in accordance with the level of respiratory tract injury in children of Eastern Siberia.

**Design of the research:**

We have examined 70 children aged 2–16 , who have food sensibilization. We divided them into 2 groups: group I (n=32) with diseases of the upper and middle respiratory tract; and group II (n=38) with diseases of the lower respiratory tract.

**Methods:**

Allergological medical history, clinical laboratory examination and immunological examination, including the determination of IgA, IgM, IgG and IgE in blood serum. In cases where causal allergens were found, elimination diets were recommended.

**Results:**

Onset of upper respiratory tract injury in group I was more often registered in children aged 0–1; in group II, it was in the 3–7 age group. Isolated food sensibilization was more often marked in group I as compared to group II. Atopic mechanisms of respiratory tract injuries were more often registered in group II children. In the course of the elimination diet, we marked positive dynamics in 100% of group I and in 75% of group II

**Conclusion:**

The most frequent allergens that cause respiratory forms of food allergy are hen eggs, cow milk, nutritive cereals, vegetables and fruit. Indices of a humoral link of immunity in the examined patients were more often registered as normal or their level is increased. Timely etiotropic therapy in the majority of cases allows for a stabilization of allergic inflammation.

The diseases of respiratory tract are still the leaders in the structure of morbidity and mortality, which causes medical and social importance of this problem. Children are the first to be recognized as a high-risk group, namely those with pathological processes in the respiratory tract. The reasons for a relapse of pathological processes in the respiratory system can be due to chronic inflammatory diseases, transitory age peculiarities of the immune system and other pathological processes of acquired or congenital genesis (1). In some cases, respiratory organs become “shocking” for the development of allergic inflammation. Food products provide humans with the first and the most important antigen exposure. That is why in children food allergy is one of the earliest and most frequent reasons of the formation of pathological process. Going forward, it determines the tempo of their development and the location of allergic inflammation (reactive factor). The question of the frequency of respiratory symptoms in food allergy is still wide open, because data on this research is minimal and it is sometimes contradictory. According to some authors, food sensibilization is present in 6–25% in children with respiratory diseases. Others say it is in 80% of cases (2, 3). Respiratory signs of a food allergy can be found in the upper and lower respiratory tract. With this in mind, the course of the food allergy can be taken from different nosological forms (rhinitis, sinusitis, tracheitis, bronchitis, etc.), and is in many cases not recognized as a cause of the disease. This results in therapy for recurrent pathological processes in the respiratory tract with antibacterial, antivirus preparations being inefficient. A food allergy, which is diagnosed on time, leads to the formation of a severe, constantly relapsing course of the disease, and the involvement of other organs and systems into the pathology process. According to some authors, the food allergy is “starting” sensibilization here in many cases. Based on its background, the spectrum of sensibilization to allergens of different groups is broadening (pollen, domestic) with ageing. Other authors believe it has the same meaning as aspiration allergy or sometimes it is more significant (4, 5). Therefore, inconsistencies in published data necessitate further research in this sphere.

The aim of our research is to determine risk factors in food allergy development, identify the characteristics of its etiologic structure, clinical immunological signs and the efficiency of elimination diet therapy in children with the diseases of upper and lower respiratory tract.

## Materials and methods

We have examined 70 children aged 2–16. Among them, 76% were in pre-school and 24% were older than 7 years. We defined 2 groups. Group I (n=32) with diseases of the upper and middle respiratory tract (rhinitis, adenoiditis and tracheitis). They belonged to the category “frequently and chronically ill children” (frequent respiratory diseases in medical history). Group II (n=38) with diseases of the lower respiratory tract (bronchial asthma). The main criteria for subjects to be involved in the research included 1) the presence of food sensibilization; 2) Group I: acute diseases of upper respiratory tract with a frequency higher than 6 times a year, the duration of the diseases over 6 months; 3) Group II: “Bronchial asthma”. In bronchial asthma, the course of the disease was registered as mild in 74% and severe in 26% of cases. In group I, the symptoms of respiratory diseases (sneezing, nasal blockage, coughing and/or temperature) were marked monthly in 63% of cases, and 6–7 times a year in 37% of cases. In group II, disease exacerbation in 53% was registered no more than 6 times per year; in 29% of cases, 2–3 times a week; and in 18% of cases, 2–3 times a month.

The research was carried out by standard clinical laboratory techniques, including medical history (onset of the disease, frequency, character of clinical signs) and results of laboratory tests. All children passed an immunological examination. The concentration of common immune globulins (A, M, G) in blood serum was estimated by G. Manchini radial immunodiffusion technique and the content of common IgE by immune enzyme analysis.

The food allergy was diagnosed after complex clinical laboratory examination of the patients taking into account the data of medical history, skin tests with food, domestic, pollen allergens, elimination and provocation tests. In cases when cause allergens were found, individual elimination diets were introduced. Their efficiency was evaluated by the dynamics of clinical signs and common conditions of a child. The duration of patient supervision in the course of diet therapy was 6–12 months. The results were regarded as excellent, when clinical symptoms disappeared; good, when the symptoms considerably decreased; and satisfactory, when there were mild improvements in clinical signs. Data processing was carried out using applied programs «Statistica 6.0».

## Results

In the development of a food allergy, genetics play a considerable role. Having analyzed risk factors in the formation of a food allergy, among them the first place belongs to atopia tainted heredity, we marked that in 69% of the examined children where their parents or relatives suffered from different allergy diseases. In group I, the frequency of hereditary taint was registered more often (80%) than in group II (58%). The nature of infant feeding and the duration of breast-feeding are extremely important in allergy development. The analysis of the data showed that even though breast feeding duration was 1 year in most children within both groups, the duration of breast-feeding was up to 3 months of age in every third child in group II (Table I). It appears to be the leading risk factor in the development of severe forms of food allergies in children, bronchial asthma in particular.

**Table I:** Duration of breast feeding in infants and time period of first manifestation of symptoms of respiratory tract disturbances in children (%)

Ages	Group I (n=32)	Group II (n=38)
Duration of breast feeding in infants		
0–3 months	0	33
0–6 months	43	0
0–12 months	41	7
Over 12 months	16	0
Time period of first manifestation of symptoms of respiratory tract disturbances		
Earlier than 1 year	40	17, p<0.01
1–3 years	35.5	33
4–7 years	13.5	42, p<0.001
Senior than 8 years	1	8

When studying allergological medical history, we revealed that the first symptoms of allergy, such as skin signs were registered in early ages in 42.5% of cases. True differences between groups I and II had not been marked. Furthermore, the percentage of this or that food intolerance in group I tended to decrease (39%) with growing-up as distinguished from group II (58%). Having analyzed the time when the first symptoms of respiratory tract disturbances appear, we marked some differences between the groups (Table I). In group I, the onset of respiratory tract disorders was registered earlier than age 1 year and there was a gradual decline with growing-up. On the contrary in group II, the percentage of lower respiratory tract disorders increased with growing-up and bronchial asthma morbidity peak relates at ages 3–7.

It is known that in food sensibilization, the allergic disorders of respiratory tract can be isolated and also they can be accompanied by disturbances to other organs. In such cases, a system allergy disease is being formed: dermatologic gastrointestinal, respiratory intestinal syndromes, atopic disease. In 83% of the cases in group I, we found comorbid complaints related to other organs in anamnesis vitae: 55% of the children complained of frequent nasal blockage, 34% had stomach pain after meals, 10% had a poor appetite and stool problems. In group II, comorbid complaints were less frequent (67%): 58% complained of frequent nasal blockage, 33% had rhinitis, 17% had headaches and 8% suffered from stomach pain after meals.

Beside hereditary traits, the sensibilization of organisms is critical to food allergy development. The results of skin tests are definitely interesting. In group I, we marked isolated food sensibilization in 36% of cases, and polyvalent (domestic, pollen) in 64% of cases. In group II, in the majority of cases (92%), we had marked polyvalent sensibilization. In group I, skin tests showed the following foods in the structure of etiologic factors: hen egg, 80%; cow milk, 62%; chicken, 56%; wheat flour, cereals (peeled barley, ground oat, buckwheat), 50%; fish, 49%; citrus, 44% cases. In group II, we had marked: hen egg, 73%; cow milk, 68%; cereals, 48%; vegetables and fruits, 47%; citrus, 36%; fish, 11% cases. The sensibilization to 4 and more foodstuffs was typical in both groups in the majority of cases (83%) and only in 17% it was found in regard to 1–3 foodstuffs. Taking into account that the said foodstuffs are consumed daily, it is not possible to state clear dependence between their consumption and exacerbations (after anamnesis data). With this in mind, a food allergy was confirmed by elimination and provocation tests in each case. In group I, the exacerbation of respiratory symptoms was caused by hen egg in 82%, cow milk in 64%, wheat flour in 55%, vegetables and fruits in 14% and fish in 9% of cases. In group II, the exacerbation of bronchial asthma was caused by hen egg in 90% of cases, cow milk in 76%, vegetables and fruits in 52%, wheat flour in 48%, meat in 14%, and nut and fish in 9% of cases.

Clinical characteristic of respiratory signs of food allergies is all-seasonal course of the disease in the majority of cases (72% in group I and 100% in group II) and gradual start of the disease. A clinical picture of a bronchial asthma attack was characterized by a pre-attack period in all of the children. Its duration was shorter in patients with an allergy to fish, nuts, citrus as compared to subjects with an allergy to milk, eggs, cereals, vegetables, and fruits. In 64% of group I, we marked the clinical symptoms, which lasted over 3 weeks, of the inefficiency of traditional methods of acute respiratory disease treatment. In bronchial asthma (group II) under associated domestic sensibilization, the disease was losing its typical dependence from the consumption of any food; the torpidity was stated in the course of the disease, which made it difficult to define the diagnosis. In comorbid pollen sensibilization, we found the seasonality in the formation of clinical symptoms of food allergy as a response to consumption of some allergy-causing food. Predominance (45%) of day–night coughing was typical in both groups; in 30% we marked night coughing; also in half of the patients coughing showed a non-productive character within the whole period of the disease.

When studying the humoral link of the immunity, we found that in subjects with respiratory signs of a food allergy that the indices of IgA, IgM, IgG are more often registered within the norm or their increased level had been marked (Table II). Only in 34% of cases did we reveal “transitory hypogammaglobulinemia of infancy” and we did not mark any true differences between groups. We marked insufficient level of IgA in the most cases. Its deficiency is known to be an unfavorable factor in the development of a food allergy.

**Table II:** Frequency of the changes in the indices of immunity humoral link in children with respiratory forms of food allergy (%)

	Hyperproduction of immunoglobulins		Norm indices		Transitory hypogammaglobulinemia	
		
Immuno globulins	Group I (n=32)	Group II (n=38)	Group I (n=32)	Group II (n=38)	Group I (n=32)	Group II (n=38)
IgA	20	43, p<0.001	43	23, p<0.01	34	30
IgM	45	53	37	23	18	23
IgG	50	37	32	40	18	23
IgE	32	53, p<0.01	68	47, p<0.01	–	–

It is well-known that the main immunological mechanism of a food allergy is the 1st type of tissue injury (reaginic). This mechanism is mediated by antibodies related to immune globulins of IgE and IgG4 class. Our research showed the increase of common IgE level in group I only in 32% cases and in Group II in 53% cases. This can prove that, with the exception of IgE-mediated mechanisms, the leading role in food allergy pathogenesis belongs to IgG4 class reagines, which are known to be less aggressive than IgE and so they, to a lesser extent, disturb the respiratory system in patients with an upper respiratory tract disorder.

Etiotropic therapy was prescribed for children with “food allergy” diagnosis (individual elimination diets) and other kinds of therapy due to the status of the patient (26% of bronchial asthma), among them stabilizers of membranes of mast cells, anti-inflammatory preparations Intal, Tilade. The period of diet therapy in most cases lasted 6 months (91%) and in 9% of cases, 12 months. In the course of elimination diet therapy, we marked positive dynamics in clinical signs in group I in 100% of cases and in group II in 75% of cases on average on days 4–5 of dieting. At later stages, group I showed excellent results in 60% of cases and good results in 40% (we marked the decrease of exacerbation number to 2–3 times per year). In group II, stabilizing of the allergy process in further time periods was registered only in 56% of cases. This data confirms a high meaning of the influence of broadening sensibilization spectrum (mostly on the account of aspiration allergens – pollen and domestic) upon the efficiency of the elimination diet therapy.

## Conclusion

In food sensibilization, the disorders of the respiratory tract are possible at any level, which is why clinical signs of food allergy show significant diversity. In the structure of etiological factors of the development of respiratory forms in food allergy, the most frequent are hen egg, cow milk, cereals, vegetables and fruits. In children with respiratory signs of a food allergy, the indices of a humoral link of immunity in most cases are registered within the norm and demonstrate increased levels. Timely conducted etiotropic therapy in most cases allows us, within a short period of time, to achieve stabilization of allergic inflammation, to decrease the frequency of retrocession, and to prevent progression of the disease under different signs including respiratory ones.
